# Soluble immune checkpoint factors reveal high-risk osteosarcoma subtypes and enable early metastasis prediction

**DOI:** 10.3389/fimmu.2025.1651051

**Published:** 2025-09-02

**Authors:** Hanqi Peng, Binghao Li, Jiameng Cui, Gege Sun, Qinchuan Wang, Yun Zhu, Sicong Wang, Huakang Tu, Xifeng Wu, Zhaoming Ye

**Affiliations:** ^1^ Center of Clinical Big Data and Analytics of the Second Affiliated Hospital and School of Public Health, Zhejiang University School of Medicine, Hangzhou, China; ^2^ Department of Orthopedics, Second Affiliated Hospital, Zhejiang University School of Medicine, Hangzhou, Zhejiang, China; ^3^ Clinical Research Center of Motor System Disease of Zhejiang Province, Hangzhou, Zhejiang, China; ^4^ Department of Surgical Oncology, Affiliated Sir Run Shaw Hospital, Zhejiang University School of Medicine, Hangzhou, Zhejiang, China; ^5^ Zhejiang Key Laboratory of Intelligent Preventive Medicine, Zhejiang, China; ^6^ National Institute for Data Science in Health and Medicine, Zhejiang University, Hangzhou, Zhejiang, China

**Keywords:** osteosarcoma, soluble immune checkpoint factor, non-specific immune cells, biomarkers, prediction model

## Abstract

**Background:**

Osteosarcoma is a rare disease, yet it is the most frequent primary malignant bone tumor, with poor survival in metastatic cases. Current PD-1 and PD-L1 checkpoint inhibitors show limited efficacy in osteosarcoma, necessitating further investigation into other immune checkpoint factors.

**Methods:**

We analyzed immune checkpoint proteins in plasma from 67 osteosarcoma patients and 50 healthy controls, examined their transcriptional levels in tumor tissues, validated the results using public databases, and elucidated potential mechanisms.

**Results:**

CD48, TIMD-4, B7-H6, CD134, B7-H5, CD47, and S100A8/A9 were significantly elevated in osteosarcoma patients, each linked to increased osteosarcoma risk. In patients who developed metastasis, CD48, B7-H2, TIMD-4, B7-H6, CD134, B7-H5, CD47, and S100A8/A9 were also elevated and correlated with higher metastasis risk. Using peripheral blood levels of these eight factors, we identified osteosarcoma immune subtypes and built an excellent predictive model for metastasis (C-index = 0.876, predicting metastasis within one year). The gene expression of these factors in tumor tissues showed an inverse correlation with metastasis compared to peripheral blood. Single-cell analysis revealed differential expression of these factors in non-specific immune cells from metastatic patients.

**Conclusion:**

Soluble immune checkpoint factors were identified as significantly associated with osteosarcoma metastasis. Using peripheral blood biomarkers, we characterized immune subtypes of osteosarcoma, and developed a predictive model for metastasis. These biomarkers may serve as potential therapeutic targets for future immunotherapy.

## 1 Introduction

Osteosarcoma, the most prevalent type of primary malignant bone tumor in humans, primarily affects adolescents, followed by elderly individuals over 60 years old (1). The 5-year survival rate for osteosarcoma patients has risen to approximately 70% (2), but for those with metastasis, it is still under 20% (3). There are currently limited clinical options to improve survival rates for metastatic osteosarcoma patients.

Soluble immune checkpoint factors are generated from membrane-bound immune checkpoint receptors or ligands through cleavage of the extracellular domain or alternative splicing (4). Soluble immune checkpoint factors are associated with the occurrence, prognosis, and treatment response of various types of cancer (4–6).

Immune checkpoint blockade therapies are increasingly applied to various solid tumors. However, common therapeutic targets such as PD-1 and PD-L1 have shown limited efficacy in osteosarcoma treatment (7–9). Current immunotherapies mainly focus on T cells, with less emphasis on exploring therapies targeting non-specific immune cells. Identifying new effective therapeutic targets is urgent, and further investigation into the clinical significance of other immune checkpoint factors in osteosarcoma is needed.

Osteosarcoma’s high heterogeneity presents unique challenges for its treatment. The differences in the tumor immune microenvironment of osteosarcoma patients, including variations in immune cell infiltration patterns and molecular expression, can significantly impact the efficacy of immunotherapy and tumor progression (10, 11). However, research on immunotyping in osteosarcoma is still in its early stages (12). Establishing immunotypes will help identify specific immunotherapeutic targets and predict patient prognosis.

In this study, we identified and validated a set of immune checkpoint-related factors as potential novel biomarkers for osteosarcoma. Immune molecular subtypes of osteosarcoma were established based on peripheral blood factor levels, which are easily accessible for collection and testing. These subtypes were subsequently used to construct an effective predictive model for osteosarcoma metastasis. We revealed the potential mechanisms by which these factors influence tumor progression by regulating the function of nonspecific immune cells.

## 2 Materials and methods

### 2.1 Participants and data acquisition

All study participants were sourced from the Healthy Zhejiang One Million People (HOPE) Cohort. A total of 67 osteosarcoma patients were selected from the bone cancer patient subcohort of the HOPE cohort, initiated in June 2020 and ongoing. These patients were newly diagnosed, pathologically confirmed cases of osteosarcoma who had not undergone any surgical or chemotherapy treatment. Fifty healthy controls were chosen from the general population subcohort of the HOPE cohort. To reduce confounding effects, osteosarcoma patients and healthy controls were matched by age (± 5 years) and sex. This research received approval from the Institutional Review Board of the Second Affiliated Hospital of Zhejiang University. All participants provided signed informed consent. Clinical data were obtained from medical records, while follow-up information was gathered through regular telephone interviews. Patients with metastases were defined as those diagnosed with metastases during follow-up visits at our hospital or confirmed through telephone follow-up. The definitions of recurrence and death were established using similar criteria. Epidemiological data were collected via face-to-face interviews using a standardized questionnaire.

Each participant had 20 mL of blood collected into three vacutainer tubes (Thermo Fisher Scientific, USA): two lavender-colored tubes containing EDTA-Na and one red-colored tube without additives. Plasma was separated from the tubes containing EDTA-Na and stored at -80°C for future use. Tumor tissue samples were collected during surgery, immediately snap-frozen, and stored at -80°C for future use.

Additionally, mRNA expression data for 39 osteosarcoma tumor tissue samples were retrieved from the Gene Expression Omnibus (GEO) dataset GSE21257. scRNA-seq data for osteosarcoma tumor tissues were obtained from the GEO dataset GSE152048.

### 2.2 Detection of soluble immune checkpoint proteins

We used the ProcartaPlex human immune checkpoint panel (Thermo Fisher, USA) in a 96-well plate format to analyze plasma samples in order to quantify immune checkpoint proteins, including CD48, B7-H2, TIMD-4, B7-H6, CD134, B7-H5, CD47, S100A8/A9, and B7-H3. The assays were conducted using the FLEXMAP 3D system (Luminex, USA) and the xPONENT^®^ 4.3 software. To reduce batch effects in the Luminex assays, we included replicate standard samples across all batches for calibration and processed all samples within a single run. A double-blind methodology was maintained throughout the detection process. The quantification procedures have been detailed in our previous study (13). The lower limit of quantification for the proteins is provided in **Supplementary Table 1**.

### 2.3 RNA extraction and gene expression quantification from tissue samples

Total RNA was isolated using the AllPrep DNA/RNA/miRNA Universal Kit (Thermo Fisher, USA), and reverse transcription was performed with HiScript III All-in-one RT SuperMix Perfect for qPCR (Vazyme, CN). cDNA amplification was then carried out using the ChamQ Universal SYBR qPCR Master Mix (Vazyme, CN) system. For each gene, two pairs of highly specific primers were initially designed, and the primer with the highest amplification efficiency was chosen for further analysis. GAPDH served as an internal control to determine the relative mRNA levels of *CD48*, *ICOSLG*, *TIMD4*, *NCR3LG1*, *TNFRSF4*, *VSIR*, *CD47*, *S100A8*, and *S100A9*. All assays were performed in triplicate.

### 2.4 Statistical analysis

Frequency and percentage [n (%)] were used to present categorical variables, while medians [interquartile range (IQR)] or mean ± standard deviation was used for continuous variables. Comparisons of categorical variables used Pearson’s χ² test, while continuous variables were compared using the t-test or Wilcoxon rank-sum test. False discovery rate (FDR) correction was applied to the *p*-values to reduce the risk of Type I errors. Pathway enrichment analysis of differential immune checkpoint proteins was conducted using Metascape (https://metascape.org) (14).

After adjusting for age, sex, alcohol consumption, smoking, hypertension, BMI, and diabetes, multivariable unconditional logistic regression was applied to estimate the relationship between each plasma immune regulatory protein and the risks of osteosarcoma incidence and metastasis. Protein levels were considered continuous variables and log-transformed to minimize skewness. Multivariable Cox proportional hazards analysis, adjusted for the same covariates, was utilized to identify independent prognostic factors for osteosarcoma.

Unsupervised consensus clustering of osteosarcoma patient samples was conducted using the R package ConsensusClusterPlus (version 1.60.0) (15). The K-means algorithm was iterated 1000 times to ensure the stability of the clustering. The optimal number of clusters (k) was determined based on the cumulative distribution function and the proportion of ambiguous clustering. Osteosarcoma patients were classified into two categories to define plasma immune regulatory protein subtypes. The distribution differences among the clusters were visualized using principal component analysis (PCA).

Survival differences were compared using the log-rank test and Kaplan-Meier curves. A multivariable Cox proportional hazards regression model incorporating plasma immune regulatory protein subtypes and clinical variables was constructed to predict metastasis at six months (180 days), one year (365 days), and two years (730 days) post-diagnosis. Tumor staging was excluded from the Cox model, as all patients had similar stages at enrollment. Model discrimination was evaluated using Harrell’s concordance index (C-index) and time-dependent receiver operating characteristic (ROC) curves. Calibration was assessed using calibration curves generated from 1,000 bootstrap resamples. Overall model performance was measured using the integrated Brier score (IBS) (16).

For scRNA-seq data processing, Seurat (version 5.1.0) was used. Cells were filtered by restricting the number of detected genes per cell, total molecule count, and mitochondrial gene expression percentage, removing empty droplets, doublets, multiplets, and low-quality cells. Batch correction was performed using the Harmony package (version 1.2.3). Data normalization was conducted using the NormalizeData function in Seurat, and the identification of the top 2,000 highly variable genes was performed with the FindVariableFeatures function, followed by centering and scaling the data. PCA was performed to determine the number of principal components needed for further analysis. Unsupervised cell clustering was carried out through the FindClusters function in Seurat (default Louvain algorithm), and two-dimensional visualization was generated using Uniform Manifold Approximation and Projection (UMAP) and t-Distributed Stochastic Neighbor Embedding (tSNE). Cell type annotation was conducted by identifying highly differentially expressed genes for each cell cluster using the FindAllMarkers function in Seurat (default non-parametric Wilcoxon rank-sum test and Bonferroni correction). Annotation was based on the top ten differential genes for each cluster and known marker genes from the literature.

All statistical analyses, except for pathway enrichment analysis, were performed in R (version 4.3.2), with all tests being two-sided and a significance threshold of 0.05.

## 3 Results

### 3.1 Patient characteristics

This study included 117 participants, consisting of 67 osteosarcoma patients and 50 healthy controls. Clinical and demographic characteristics are presented in **Table 1**. The median age difference between the groups was 3.5 years. Among all participants, approximately 80% were male, with almost no history of smoking or alcohol consumption, and most had normal BMI, with minimal cases of diabetes or hypertension. Within the patient group, 14 cases (20.9%) experienced metastasis, 4 cases (6.0%) resulted in mortality, and 3 cases (4.5%) showed recurrence. The median follow-up time was 394 days (range: 11–1018).

**Table 1:** Host characteristics of healthy controls and osteosarcoma patients.

Variables	Case (n=67)	Control(n=50)	*p*
Age, median (IQR)	19.00 (12.50, 31.00)	22.50 (20.25, 45.25)	<0.001
Age group, n (%)			
<60	59 (88.06)	42 (84.00)	0.72
>=60	8 (11.94)	8 (16.00)	
Sex, n (%)			
male	51 (76.12)	41 (82.00)	0.59
female	16 (23.88)	9 (18.00)	
Smoke, n (%)			
yes	18 (26.87)	13 (26.53)	1
no	49 (73.13)	36 (73.47)	
Drink group, n (%)			
no	53 (79.10)	32 (65.31)	0.15
yes	14 (20.90)	17 (34.69)	
BMI, median (IQR)	20.65 (17.51, 23.19)	22.09 (20.21, 25.45)	4.00E-03
BMI group, n (%)			
<25	57 (85.07)	36 (72.00)	0.13
>=25	10 (14.93)	14 (28.00)	
Hypertension, n (%)			
no	66 (98.51)	45 (90.00)	0.10
yes	1 (1.49)	5 (10.00)	
Diabetes, n (%)			
no	66 (98.51)	50 (100.00)	1
yes	1 (1.49)	0 (0.00)	

### 3.2 Soluble immune checkpoint proteins associated with osteosarcoma risk

Luminex multiplex assays were performed to measure 10 soluble immune checkpoint proteins in all participants. Since S100A8 and S100A9 frequently exist as the S100A8/A9 complex in plasma, we measured this complex (17). The B7-H3 assay was excluded due to an invalid standard curve. Protein levels are detailed in **Table 2**.

**Table 2:** Circulating levels of immune checkpoint proteins across all study subjects.

Proteins	Case	Control	*p*
	Median (IQR) pg/ml	Median (IQR)pg/ml	
CD48	250.14 (80.70, 497.19)	100.72 (80.94, 142.18)	**6.40E-03**
B7-H2	239.37 (148.03, 280.71)	184.39 (158.66, 218.78)	0.10
TIMD-4	833.63 (557.38, 1037.65)	397.92 (325.13, 478.66)	**<0.001**
B7-H6	1163.41 (280.85, 2109.09)	365.67 (266.43, 497.74)	**1.80E-03**
CD134	47.90 (25.12, 105.57)	29.05 (22.44, 35.40)	**<0.001**
B7-H5	145.87 (49.54, 238.34)	56.64 (45.53, 68.04)	**<0.001**
CD47	53.89 (14.69, 67.84)	12.82 (9.76, 15.81)	**<0.001**
S100A8/A9	1482.64 (849.06, 2315.93)	804.73 (678.14, 1035.83)	**<0.001**

Significant *p* values in bold font.

**Figure 1:**
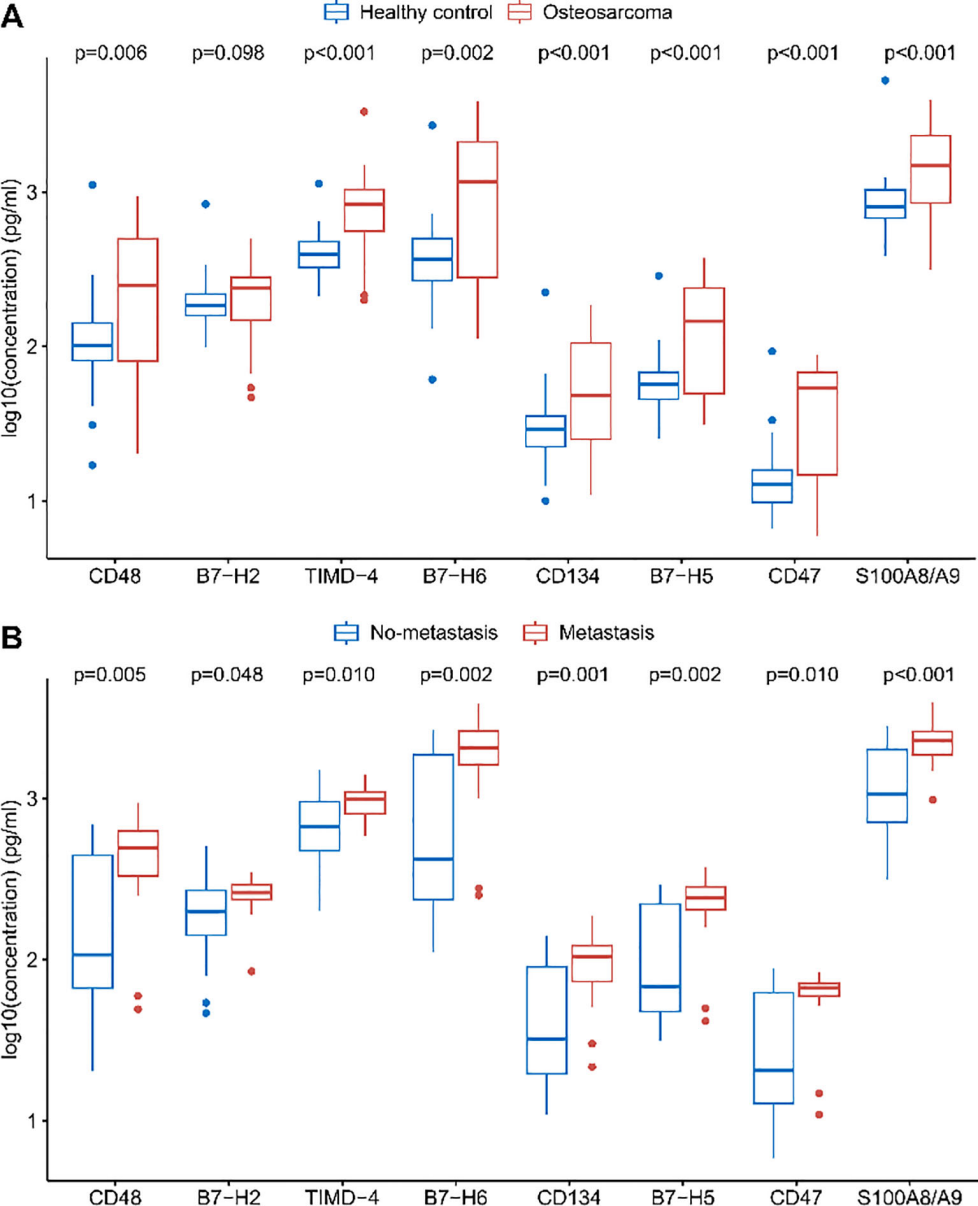
Differences in plasma levels of immune checkpoint proteins. **(A)** Comparison of soluble immune checkpoint protein levels between healthy controls and osteosarcoma patients. **(B)** Comparison of soluble immune checkpoint protein levels between osteosarcoma patients without metastasis and those with metastasis. FDR correction was applied to the *p*-values.

With the exception of B7-H2, all measured immune checkpoint proteins—CD48, TIMD-4, B7-H6, CD134, B7-H5, CD47, and S100A8/A9—were significantly elevated in osteosarcoma patients compared to healthy controls (**Figure 1A**, p < 0.05). Multivariate unconditional logistic regression analysis showed that higher levels of these protein markers were significantly linked to an increased risk of osteosarcoma (**Table 3**).

**Table 3:** Associations between soluble immune checkpoint proteins and the risk of osteosarcoma occurrence and metastasis.

Proteins	Case vs. control		Metastasis vs. no-metastasis	
	OR (95%CI)^a^	*p* ^b^	OR (95%CI)^a^	*p* ^b^
CD48	1.87 (1.22-2.99)	**6.54E-03**	1.62 (1.06-2.57)	**0.04**
B7-H2	1.14 (0.76-1.72)	0.54	1.13 (0.74-1.74)	0.58
TIMD-4	6.60 (3.43-14.93)	**2.95E-06**	3.22 (1.95-5.79)	**1.73E-04**
B7-H6	2.33 (1.48-3.88)	**7.84E-04**	1.71 (1.12-2.69)	**0.02**
CD134	2.19 (1.40-3.60)	**1.32E-03**	1.77 (1.15-2.81)	**0.02**
B7-H5	3.23 (1.92-5.96)	**1.07E-04**	2.26 (1.43-3.77)	**2.22E-03**
CD47	4.22 (2.41-8.31)	**1.57E-05**	2.79 (1.73-4.81)	**3.05E-04**
S100A8/A9	2.54 (1.60-4.25)	**3.13E-04**	1.93 (1.25-3.09)	**8.20E-03**

a Adjusted by age, sex, smoking, alcohol consumption, BMI, hypertension and diabetes. Tumor staging was not included as all patients had the same stage at enrollment.

b FDR-correction was applied.

Significant *p* values in bold font.

Levels of eight immune checkpoint proteins (CD48, B7-H2, TIMD-4, B7-H6, CD134, B7-H5, CD47, and S100A8/A9) were significantly elevated in metastatic patients compared to those without metastasis (**Figure 1B**, p < 0.05). Further analysis indicated that elevated levels of these markers, with the exception of B7-H2, were significantly associated with a higher risk of metastasis in osteosarcoma patients (**Table 3**).

Pathway enrichment analysis of the differentially expressed immune checkpoint proteins identified four significantly enriched pathways: GO:0051251 (positive regulation of lymphocyte activation), GO:0046649 (lymphocyte activation), GO:0050729 (positive regulation of inflammatory response), and R-HSA-1280218 (Adaptive Immune System). All pathways had -log10(P) > 1.3 (*p* < 0.05) (**Supplementary Figure 1**), suggesting that these proteins are closely associated with lymphocyte activation and inflammatory processes.

### 3.3 Osteosarcoma immune subtypes based on soluble immune checkpoint proteins

**Figure 2:**
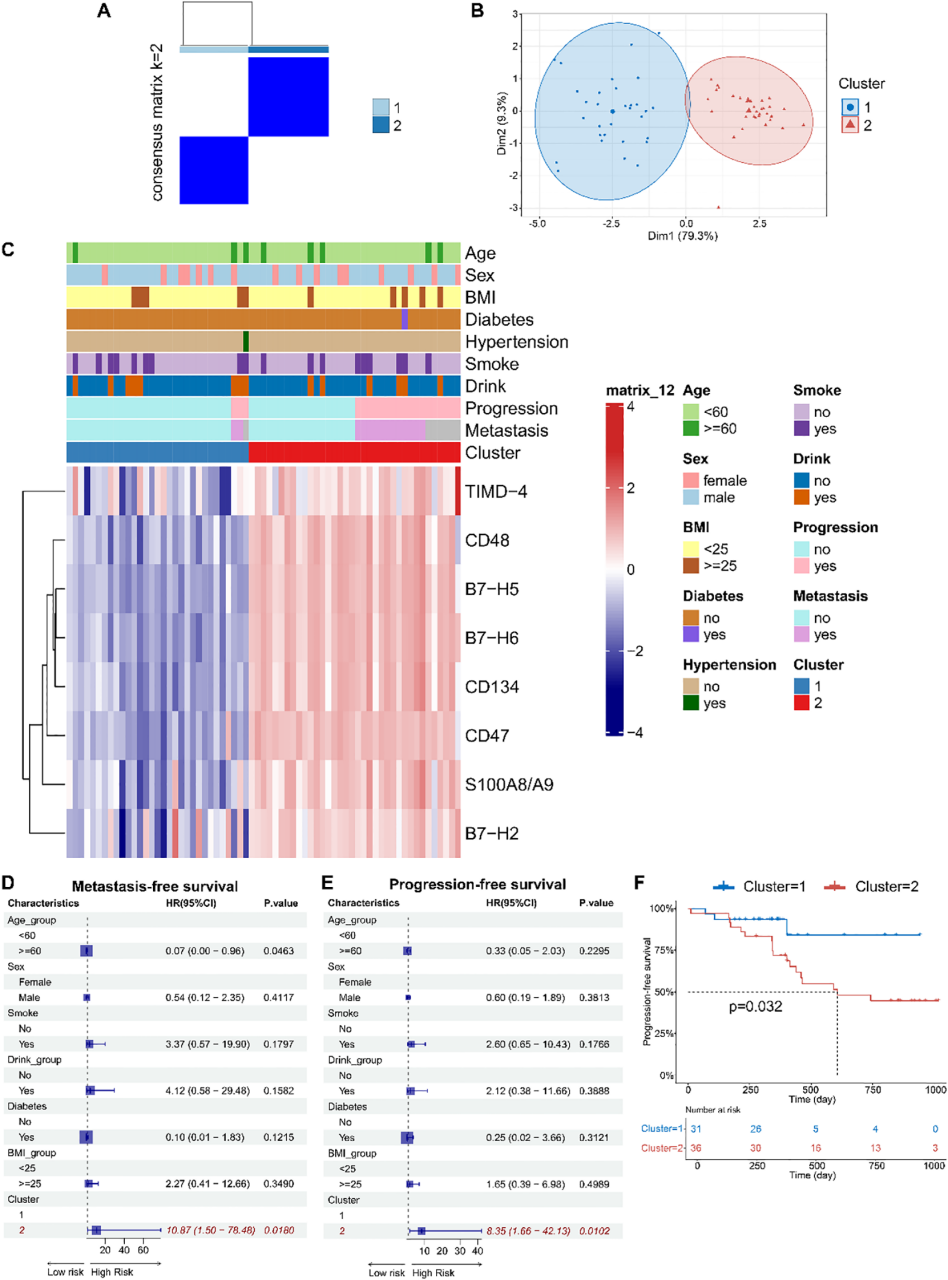
Based on peripheral blood immune checkpoint protein levels, osteosarcoma immune subtypes were identified, which were found to be associated with patient prognosis. **(A)** Consensus clustering matrix for k=2 based on the plasma levels of immune checkpoint proteins by unsupervised consensus clustering method (k-means). **(B)** PCA plot showing clear separation between the two clusters. **(C)** Heatmap displaying the association between clusters and clinical characteristics, including age, gender, BMI, diabetes, hypertension, smoking, drinking, progression, metastasis, and immune subtypes. **(D, E)** Forest plots of multivariable Cox regression analyses for osteosarcoma metastasis and progression, with covariates including age, gender, smoking, drinking, diabetes, BMI, and immune subtypes. The vertical dashed line represents HR = 1.0. The solid squares and horizontal lines represent HRs and 95% CIs. **(F)** Kaplan-Meier curves indicating that patients with immune subtype II had significantly shorter PFS compared to those with immune subtype I.

Unsupervised consensus clustering of osteosarcoma patients based on the levels of eight identified soluble immune checkpoint proteins (CD48, B7-H2, TIMD-4, B7-H6, CD134, B7-H5, CD47, S100A8/A9) classified patients into two clusters (**Figure 2A**, **Supplementary Figure 2**, k = 2): 31 in cluster 1 and 36 in cluster 2. Principal component analysis demonstrated a clear separation between these clusters (**Figure 2B**). Immune checkpoint protein levels were lower in cluster 1 compared to cluster 2, leading to their classification as Osteosarcoma Immunity Type I (cluster 1) and Osteosarcoma Immunity Type II (cluster 2). Clinical features did not significantly differ between these immune subtypes (**Figure 2C**, **Supplementary Table 2**).

Multivariate Cox regression models indicated that soluble immune checkpoint protein subtypes were strong predictors of metastasis (HR = 10.87, 95% CI: 1.50–78.48) and progression-free survival (PFS) (HR = 8.35, 95% CI: 1.66–42.13) (**Figures 2D, E**). Kaplan-Meier analysis showed that Type II had significantly shorter PFS compared to Type I (log-rank *p* = 0.032) (**Figure 2F**).

### 3.4 Prediction models for osteosarcoma metastasis

**Figure 3:**
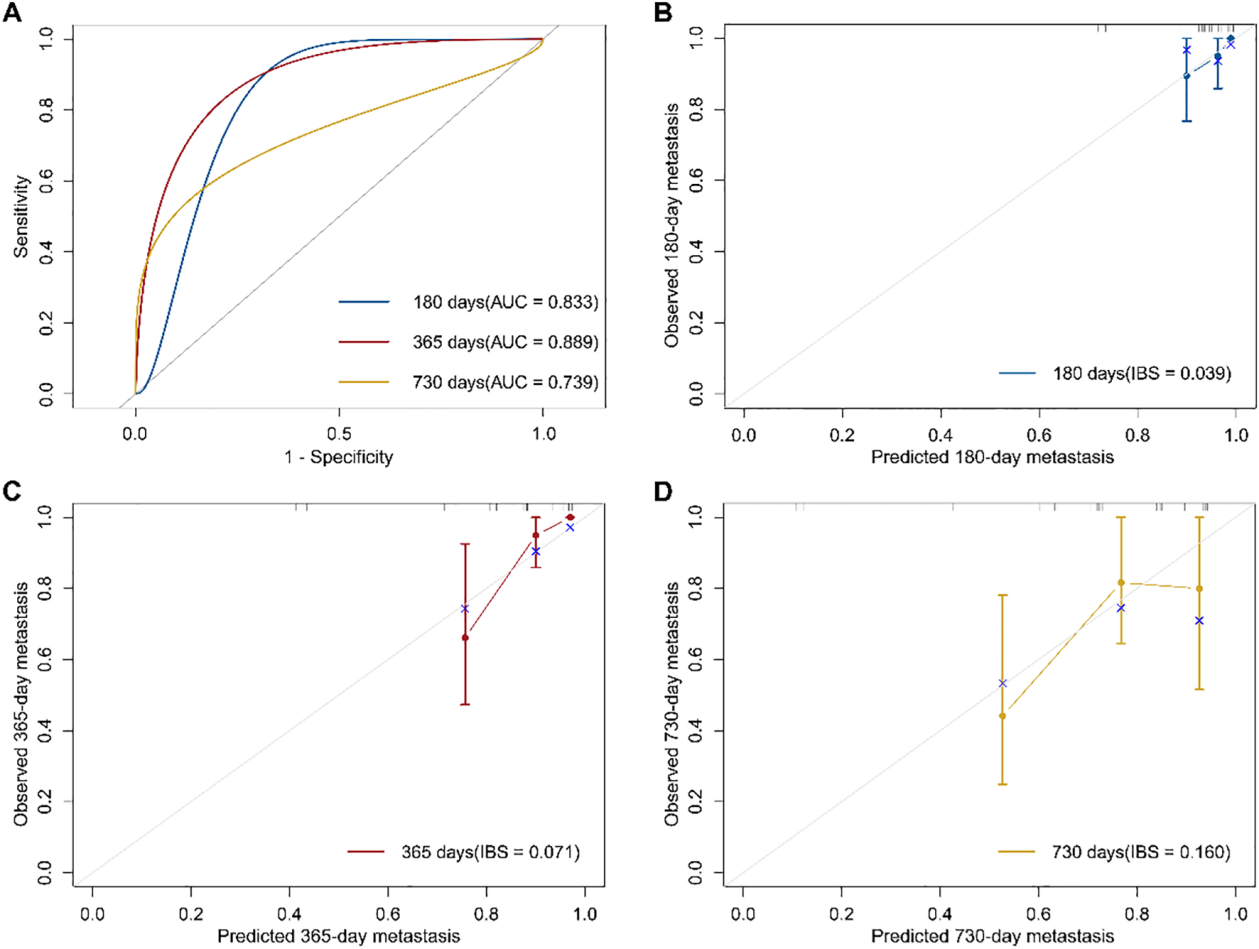
Osteosarcoma metastasis prediction model based on immune subtypes and its performance evaluation. **(A)** Receiver operating characteristic (ROC) curves of the prognostic model at 180, 365, and 730 days, with the area under the curve (AUC) indicating model performance. **(B–D)** Calibration curves of the prognostic model at 180, 365, and 730 days. The Integrated Brier Score (IBS) was used to quantify prediction error, with the gray line representing the ideal reference line.

Using soluble immune checkpoint protein subtypes and clinical variables (age, sex, smoking, alcohol consumption, BMI, hypertension, and diabetes), we constructed a multivariable Cox proportional hazards regression model to predict metastasis in osteosarcoma patients (**Figure 3A**). Tumor staging was excluded as all patients were at the same stage at enrollment. The C-index of the model at six months (180 days), one year (365 days), and two years (730 days) was 0.902, 0.876, and 0.648, respectively. The model demonstrated strong discriminative ability throughout the follow-up period, with superior performance at six months and one year. The area under the ROC curve (AUC) at six months, one year, and two years was 0.833, 0.889, and 0.739, respectively, while the IBS was 0.039, 0.071, and 0.160. Calibration curves for predicting metastasis at six months, one year, and two years showed that the predicted outcomes closely aligned with the observed results (**Figures 3B-D**). Decision curve analysis (**Supplementary Figure 3**) showed that, within the threshold probability range of 10% to 70%, the 1-year and 2-year models yielded greater net benefit than the ‘treat-all’ and ‘treat-none’ strategies, suggesting their clinical utility in identifying patients at high risk of metastasis. The 1-year model demonstrated the widest range of clinical benefit, indicating broader applicability. These findings suggest that our model, incorporating soluble immune checkpoint protein subtypes, is a valuable tool for predicting osteosarcoma metastasis.

### 3.5 The transcription levels of soluble immune checkpoint factors in osteosarcoma tumor tissues were associated with the metastasis of osteosarcoma

**Figure 4:**
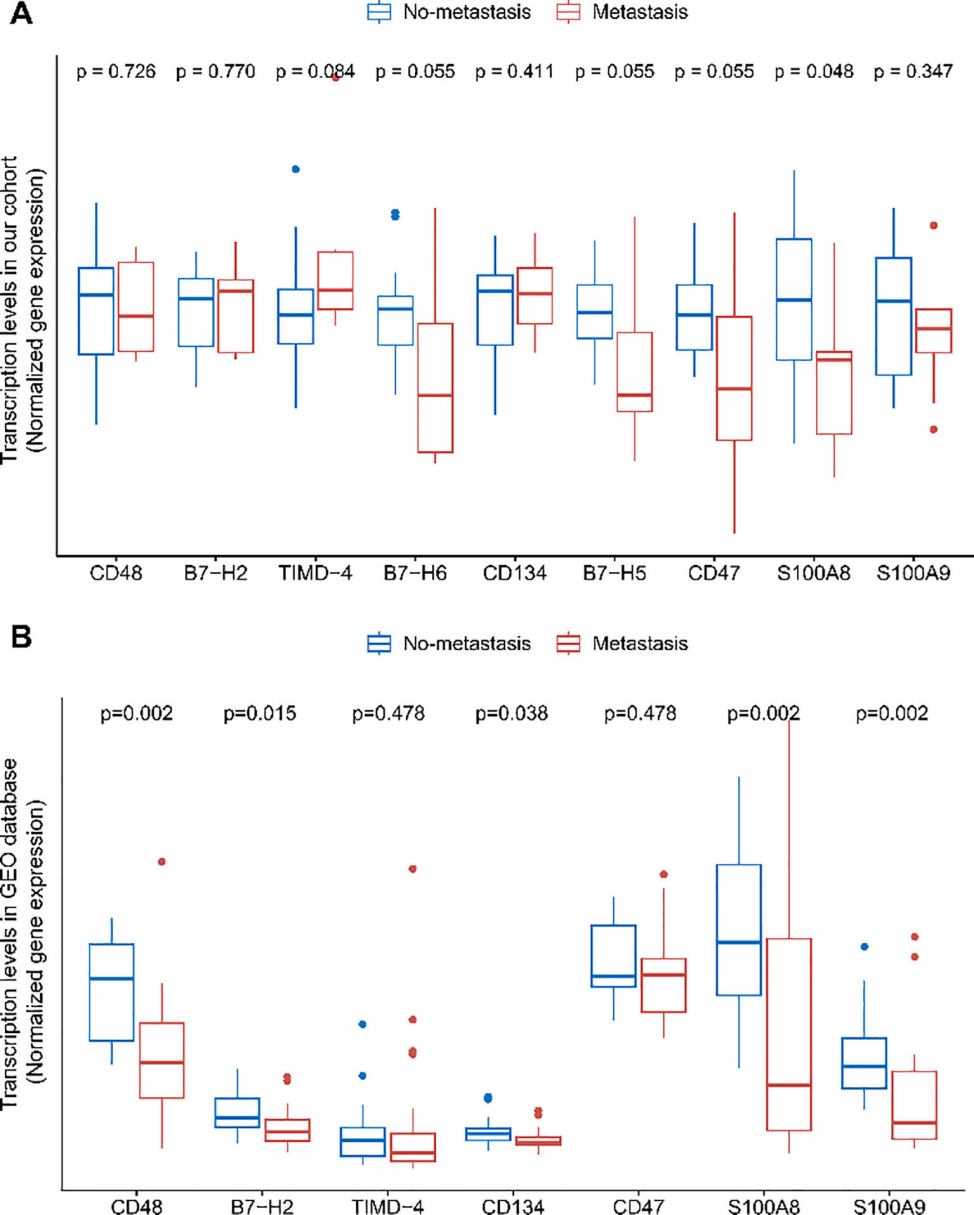
Differences in immune checkpoint protein transcript levels in osteosarcoma tumor tissues. **(A)** Comparison of immune checkpoint protein transcript levels between no-metastatic and metastatic osteosarcoma patients' tumor tissues in the HOPE cohort. **(B)** Comparison of immune checkpoint protein transcript levels between no-metastatic and metastatic osteosarcoma patients' tumor tissues in the GEO dataset GSE21257. FDR correction was applied to the p-values.

To further investigate the relationship between soluble immune checkpoint proteins and osteosarcoma and to preliminarily explore their mechanisms of action, we collected tumor tissues from 24 osteosarcoma patients (9 with metastasis and 15 without metastasis) out of the 67 who underwent plasma testing. We analyzed the expression levels of genes encoding soluble immune checkpoint proteins, including CD48, B7-H2, TIMD-4, B7-H6, CD134, B7-H5, CD47, S100A8, and S100A9 (genes: *CD48*, *ICOSLG*, *TIMD4*, *NCR3LG1*, *TNFRSF4*, *VSIR*, *CD47*, *S100A8*, *S100A9*) in these tumor tissues. Expression levels of all nine genes were observed to be lower in tumor tissues from metastatic patients compared to non-metastatic patients (**Figure 4A**). Although only the difference in S100A8 reached statistical significance, a clear trend of reduced expression was evident for the other genes. This lack of statistical significance may result from the limited sample size, given the rarity of osteosarcoma.

To validate these findings, we analyzed the expression levels in tumor tissues from 39 osteosarcoma patients (20 with metastasis and 19 without metastasis) available in the GEO database. Since the database did not include data on B7-H6 and B7-H5 expression, these genes were excluded from the analysis. We found that the expression levels of CD48, B7-H2, S100A8, and S100A9 were significantly lower in metastatic patients, with differences reaching statistical significance (**Figure 4B**). The expression levels of other soluble immune checkpoint protein genes also showed a noticeable decrease in metastatic patients. Interestingly, this contrasts with the findings observed in peripheral blood.

### 3.6 Identification of osteosarcoma immune subtypes in tumor tissues

**Figure 5:**
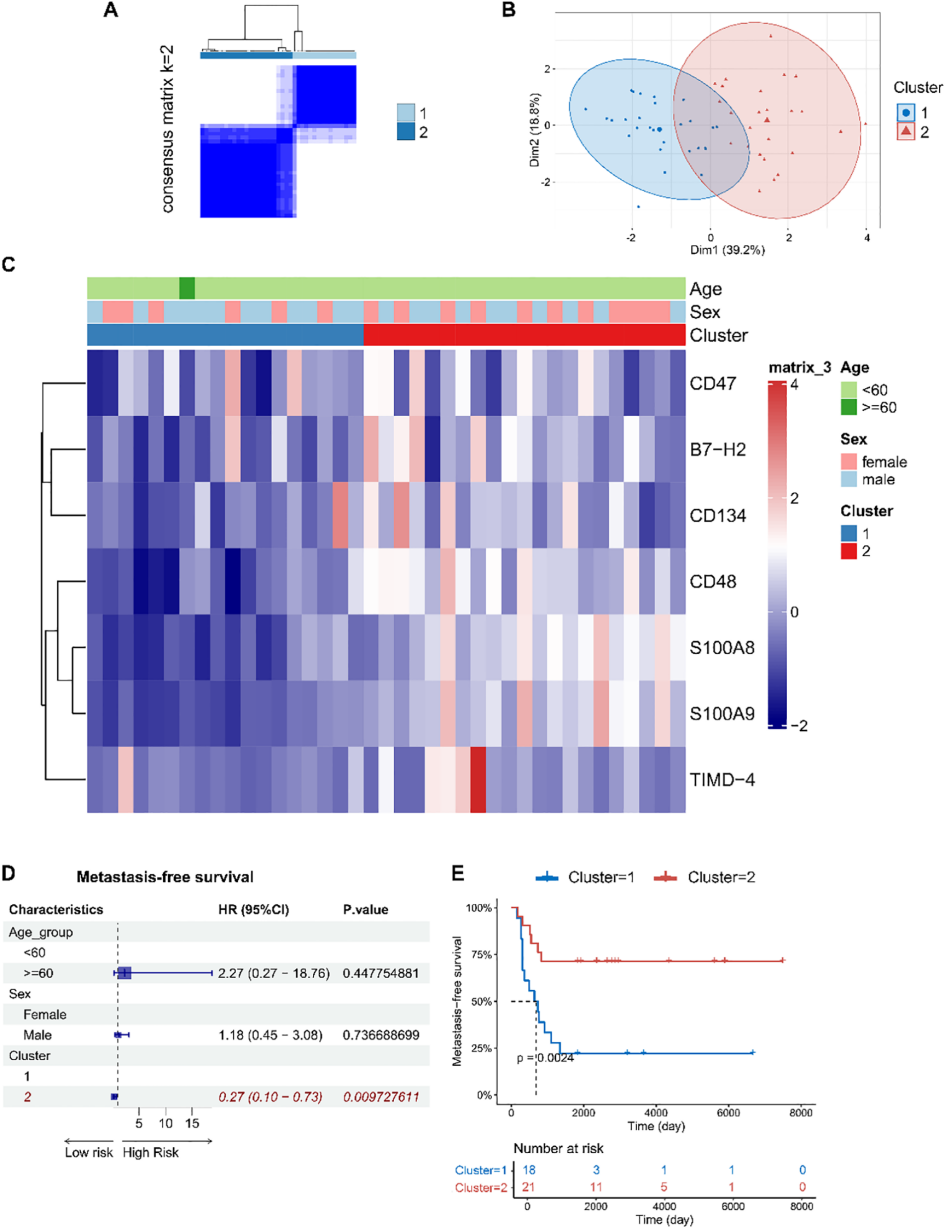
Immune subtypes of osteosarcoma were identified based on the expression levels of immune checkpoint factor genes in tumor tissues, revealing associations with patient prognosis. **(A)** Consensus clustering matrix for k=2 based on the expression levels of immune checkpoint factor genes by unsupervised consensus clustering method (k-means). **(B)** PCA plot showing clear separation between the two clusters. **(C)** Heatmap displaying the association between clusters and clinical characteristics, including age, gender, and immune subtypes. **(D)** Forest plots of multivariable Cox regression analyses for osteosarcoma metastasis, with covariates including age, gender, and immune subtypes. The vertical dashed line represents HR = 1.0. The solid squares and horizontal lines represent HRs and 95% CIs. **(E)** Kaplan-Meier curves indicating that patients with immune subtype I had significantly shorter metastasis-free survival compared to those with immune subtype II.

Based on the expression levels of CD48, B7-H2, TIMD-4, CD134, CD47, S100A8, and S100A9 (genes: *CD48*, *ICOSLG*, *TIMD4*, *TNFRSF4*, *CD47*, *S100A8*, and *S100A9*), we performed unsupervised consensus clustering of osteosarcoma patients in the GSE21257 dataset and identified two distinct immune subtypes: osteosarcoma immune subtype I (cluster 1, n = 18) and osteosarcoma immune subtype II (cluster 2, n = 21) (**Figures 5A–C**).

Interestingly, patients classified as osteosarcoma immune subtype I exhibited a higher risk of metastasis and shorter metastasis-free survival (log-rank *p* = 0.002) (**Figures 5D, E**), which is in contrast to the findings observed in peripheral blood.

### 3.7 Gene expression levels of soluble immune checkpoint factors in immune cells

To further explore the potential mechanisms underlying our findings, we analyzed scRNA-seq data from tumor tissues of 11 osteosarcoma patients in the GEO database. Batch effect correction was performed on the data (**Supplementary Figure 4**). Unbiased clustering of the cells identified 15 major cell clusters (**Supplementary Figure 5A**, **Supplementary Table 3**). All immune cell populations were extracted and subsequently re-clustered, resulting in 12 distinct immune cell types (**Supplementary Figure 5B**, **Supplementary Table 4**). Expression differences of representative marker genes within these cell populations were quantitatively assessed and presented, with biological annotations applied (**Supplementary Figure 5C**). We calculated the proportions of various immune cell types in different prognostic groups (**Supplementary Figure 5D**).

**Figure 6:**
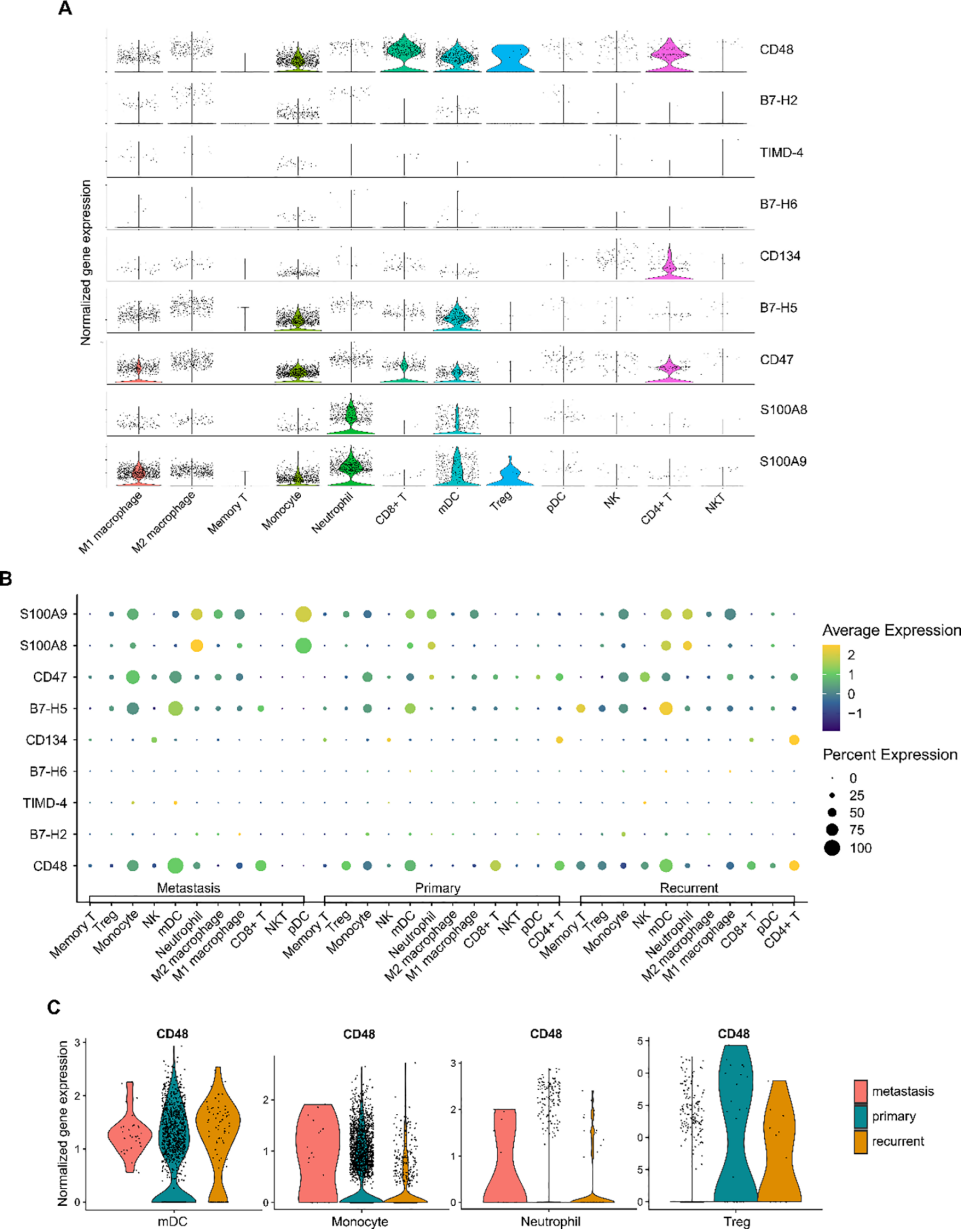
Expression of immune checkpoint protein genes in the osteosarcoma tumor immune microenvironment. **(A)** Violin plot showing the expression levels of immune checkpoint protein genes across different immune cell types. **(B)** Dot plot illustrating the differences in immune checkpoint gene expression among immune cells in metastatic, primary, and recurrent osteosarcoma tumor tissues. The size of the dots represents the percentage of cells expressing a particular gene, while the color gradient indicates the average expression level, with yellow representing high expression and blue representing low expression. **(C)** Taking CD48 as an example, violin plots show the differential expression of CD48 gene in different immune cell types and prognostic groups.

We quantified the expression of these factor genes in immune cells (**Figure 6A**) and analyzed their differential expression across various immune cell types among different prognostic groups (**Figures 6B, C**, **Supplementary Table 5**, **6**). Comparing metastatic patients with primary patients, we observed a significant elevation in the expression of the S100A8 gene in M1 macrophages in metastatic patients (p=0.003). In M2 macrophages, the expression of B7-H5, CD47, S100A8, and S100A9 was significantly upregulated in metastatic patients (p=0.037, p=0.007, p=0.003, p<0.001, respectively). For mDCs, the expression of CD48, TIMD-4, and CD47 was markedly increased in metastatic patients (p=0.022, p<0.001, p=0.012, respectively), while the expression of S100A8 and S100A9 was significantly reduced (p=0.005, p=0.012, respectively). In memory T cells, metastatic patients showed a significant downregulation of S100A9 expression (p<0.001). Additionally, in monocytes, the expression of CD48, TIMD-4, S100A8, and S100A9 was upregulated in metastatic patients (p=0.041, p=0.009, p<0.001, p=0.008, respectively). In neutrophils, the expression of CD48 and S100A8 was also elevated in metastatic patients (p<0.001, p=0.019, respectively). However, CD48 expression was significantly reduced in Tregs (p<0.001), which may impact their immunosuppressive function.

In conclusion, we reveal the relationship between the expression levels of immune checkpoint factor genes across various immune cells and osteosarcoma metastasis.

## 4 Discussion

In this study, we identified and validated a set of soluble immune checkpoint proteins associated with the progression of osteosarcoma, which could serve as novel biomarkers for the disease. By evaluating the levels of these accessible peripheral blood biomarkers, we classified osteosarcoma patients into immune subtypes. Using these subtypes in combination with clinical variables, we developed a metastasis prediction model that was successfully applied to osteosarcoma patients. Single-cell analysis further revealed that these biomarkers were closely associated with non-specific immune cells in osteosarcoma, potentially influencing tumor metastasis by modulating the function and abundance of these cells within the tumor microenvironment. Previous studies have not jointly assessed immune checkpoint factors associated with non-specific immune cells for constructing immune molecular subtypes and prognostic models. Our findings suggest that these biomarkers could serve as promising therapeutic targets for future immunotherapy in osteosarcoma.

Interestingly, we found that elevated plasma levels of these immune checkpoint proteins were associated with poor prognosis in osteosarcoma, while the transcriptional levels of their corresponding genes in tumor tissues exhibited an opposite correlation with prognosis. Single-cell analysis of osteosarcoma tissue indicated that this discrepancy might stem from differences in the tumor immune microenvironment among patient groups with differing prognoses. Further research is needed to explore this phenomenon. For example, elevated S100A8 protein levels in plasma result from its abundant secretion by neutrophils, macrophages, and monocytes, indicating systemic inflammation or enhanced metastatic potential, and are thus associated with poorer prognosis. In contrast, S100A8 mRNA expression in tumor tissues is lower, possibly due to downregulation by tumor cells. Since tumor cells constitute a large proportion of the tumor tissue, their reduced expression can obscure the contribution of infiltrating immune cells, leading to an overall decrease in observed expression.

Additionally, we observed a significant infiltration of Tregs in the tumor tissues of metastatic patients compared to those with primary osteosarcoma. This highly immunosuppressive microenvironment likely supports tumor cell survival and proliferation.

In metastatic patients, we noted increased expression of CD48 in mDCs, monocytes, and neutrophils, while its expression was reduced in Tregs. Studies have shown that CD48 has a bidirectional regulatory effect on immune cells, with moderate expression enhancing immune cell activation and function, while overexpression exerts an inhibitory effect (18, 19). This suggests that overexpression of CD48 may suppress the activity of mDCs, monocytes, and neutrophils, enabling tumor cells to evade immune surveillance. Previous research has demonstrated that CD48 can bind to growth differentiation factor 15 (GDF15), enhancing the immunosuppressive function of Tregs (20). However, we found that CD48 expression was decreased in Tregs from metastatic tumor tissues, suggesting that although the proportion of Tregs increased, their immunosuppressive capacity may be diminished in the metastatic tumor microenvironment.

B7-H2 (ICOSL) regulates T cell activation by binding to ICOS on T cells (21, 22). B7-H2 is predominantly expressed on antigen-presenting cell (23). We found elevated plasma B7-H2 but decreased tumor tissue expression in metastatic patients, possibly due to fewer antigen-presenting cells in tumor tissues. Through the B7-H2-ICOS axis, Tregs may inhibit effector T cell activity in tumors, facilitating immune evasion (24). Metastatic tumors showed higher Treg proportions, suggesting a key mechanism by which B7-H2 contributes to osteosarcoma metastasis.

We found that TIMD-4 expression was elevated in mDCs and monocytes of metastatic patients compared to primary patients. TIMD-4, also known as TIM-4, is predominantly expressed on antigen-presenting cells, especially dendritic cells. Increased TIMD-4 expression can enhance the antigen-presenting capacity of these cells (25, 26). Furthermore, TIMD-4 acts as a ligand for TIM-1, which is primarily expressed on T cells; the TIM-4-TIM-1 interaction promotes T cell proliferation (27). In our study, the elevated TIMD-4 levels in mDCs and monocytes of metastatic patients may enhance T cell proliferation through the TIMD-4-TIM-1 pathway. Correspondingly, we observed higher proportions of memory T cells and Tregs in the tumor tissues of metastatic patients compared to primary patients.

B7-H6 is primarily expressed in tumor cells. Silencing B7-H6 can increase tumor cell apoptosis, and B7-H6-specific CAR-T cell therapy has shown promising results both *in vitro* and *in vivo (* 28, 29). In our study, B7-H6 levels were elevated in the plasma of metastatic osteosarcoma patients, while its transcriptional levels in tumor tissue were reduced. This may indicate that B7-H6-expressing tumor cells have migrated into the peripheral blood, facilitating distant metastasis in osteosarcoma.

We found that CD134 is predominantly expressed on CD4+ T cells, where it plays a costimulatory role in regulating their proliferation, survival, and activation (30, 31). In metastatic osteosarcoma patients, we observed elevated CD134 levels in plasma but decreased transcriptional levels in tumor tissues. This finding suggests that a significant number of CD134-expressing CD4+ T cells may have migrated from the tumor tissue into the bloodstream.

Research on other tumor types has shown that B7-H5 is highly expressed in infiltrating immune cells within tumor tissues, where it typically inhibits T cell activation and proliferation by binding to its receptor. Blocking B7-H5 with monoclonal antibodies has been shown to effectively suppress tumor growth (32, 33). However, B7-H5 can also stimulate T cell proliferation by binding to the CD28H receptor on T cells, slowing pancreatic tumor growth (34). Additionally, B7-H5 can promote CD8+ T cell infiltration in breast cancer tumor tissues, enhancing antitumor immunity (35). These findings suggest that the effects of B7-H5 in tumor growth and metastasis may vary across different cancer types. In osteosarcoma, B7-H5 was predominantly expressed in monocytes and mDCs. Metastatic patients exhibited elevated B7-H5 levels in both plasma and tumor-infiltrating M2 macrophages, which may suppress T cell function and proliferation, thereby facilitating immune evasion and promoting metastasis.

CD47 functions as a “don’t eat me” signal, with its high expression in tumor cells inhibiting T cell function through the CD47-SIRPα pathway and promoting immune evasion (36). In our study, elevated CD47 levels were observed in the plasma of metastatic osteosarcoma patients, suggesting a role in driving macrophage polarization toward the M2 phenotype. This polarization likely enhances the immunosuppressive environment and facilitates tumor metastasis (37).

S100A8 is predominantly expressed in bone marrow-derived immune cells, particularly neutrophils and monocytes (38–40). S100A8 contributes to the tumor microenvironment by binding to Toll-like receptor 4 (TLR4) and the Receptor for Advanced Glycation End-products (RAGE), activating NF-κB and inducing the production of pro-inflammatory cytokines. This promotes tumor-associated inflammation, a critical factor in tumor initiation, progression, and immune evasion (41, 42). Moreover, S100A8 can drive monocyte differentiation into M2 macrophages, further sustaining the immunosuppressive tumor microenvironment and facilitating metastasis (43). In our study, S100A8 expression was increased in M1 macrophages, M2 macrophages, monocytes, and neutrophils of metastatic patients, while its expression in mDCs was reduced. Additionally, S100A8 levels were elevated in the plasma of metastatic osteosarcoma patients, while its transcriptional levels in tumor tissues were decreased. This discrepancy may reflect the migration of M1 macrophages, M2 macrophages, monocytes, and neutrophils into the peripheral blood of metastatic patients. S100A9 exhibits similar expression patterns and functions to S100A8.

In addition, the C-index for metastasis prediction notably decreased at 2 years. The potential causes for the drop may lie in reduced sample size, time-dependent risk factor variation. First, the osteosarcoma cases in this study are limited (N=67). The number of observed metastasis events decreases, while the proportion of censored individuals or those without metastasis increases, leading to changes in the composition of evaluable samples. This results in unstable model fitting and reduced predictive accuracy. Second, predictive factors may change over time, whereas the model is based on baseline data. Early metastasis is likely driven by baseline characteristics, which may explain the better predictive performance in the short term. In contrast, the occurrence of late metastasis may be influenced by environmental factors or changes in lifestyle. In future, regular monitoring of plasma immune checkpoint factor levels could be considered to capture their dynamic changes over time.

This study has several strengths. First, we identified and validated novel biomarkers for osteosarcoma. Second, we constructed an effective predictive model for osteosarcoma metastasis and immune molecular subtypes based on peripheral blood factor levels, which are convenient for sampling and testing. Furthermore, we utilized scRNA-seq analysis to reveal differences in the transcriptional levels of immune checkpoint factors in immune cells within tumor tissues between metastatic and primary osteosarcoma patients. This study has certain limitations. First, the sample size, particularly for metastatic patients, was relatively small. Due to the rarity of osteosarcoma, we were unable to obtain additional samples at this time. However, we employed a robust study design with strict matching to control for confounding factors and supplemented our analysis using public databases. Second, we did not perform single-cell analysis of peripheral blood in osteosarcoma patients.

## Data Availability

The original contributions presented in the study are included in the article/**Supplementary Material**. Further inquiries can be directed to the corresponding authors.
