# Supplementary material for: Soluble immune checkpoint factors reveal high-risk osteosarcoma subtypes and enable early metastasis prediction
Source: Front Immunol. 2025 Sep 2;16:1651051. doi: 10.3389/fimmu.2025.1651051 (PMC12436352; doi:10.3389/fimmu.2025.1651051)

Supplementary Material

# Supplementary Tables

| **Supplementary table 1:**  Lower limits of quantification (LLOQ) of analyte (data from ThermoFisher ^a^). | |
| --- | --- |
| Analyte | LLOQ (pg/ml) |
| B7-H6 | 119 |
| CD134 (OX40) | 7.52 |
| CD276 (B7-H3) | 571 |
| CD47 (IAP) | 6.13 |
| CD48 (BLAST-1) | 28 |
| ICOS Ligand (B7-H2) | 9.03 |
| S100A8/A9 | 61 |
| TIMD-4 | 61 |
| VISTA (B7-H5) | 8.64 |
| ^a^ https://assets.thermofisher.com/TFS-Assets/LSG/certificate/Certificates-of-Analysis/329444-003_EPX090-15820-901.pdf | |

| **Supplementary table 2:**  Patient characteristics across different immune subtypes of osteosarcoma. | | | |
| --- | --- | --- | --- |
| Variables | Immunity type I (n=31) | Immunity type II (n=36) | p |
| Age, median (IQR) | 20.00 (11.00, 34.00) | 18.500(13.00, 28.25) | 0.55 |
| Age group, n (%) |  |  |  |
| <60 | 28 (90.32) | 31 (86.11) | 0.88 |
| >=60 | 3 (9.68) | 5 (13.89) |  |
| Sex, n (%) |  |  |  |
| male | 24 (77.42) | 27 (75.00) | 1 |
| female | 7 (22.58) | 9 (25.00) |  |
| Smoke, n (%) |  |  |  |
| yes | 9 (29.03) | 9 (25.00) | 0.92 |
| no | 22 (70.97) | 27 (75.00) |  |
| Drink group, n (%) |  |  |  |
| no | 23 (74.19) | 30 (83.33) | 0.54 |
| yes | 8 (25.81) | 6 (16.67) |  |
| BMI, median (IQR) | 18.38 (16.77, 23.50) | 20.70 (18.44, 22.56) | 0.16 |
| BMI group, n (%) |  |  |  |
| <25 | 26 (83.87) | 31 (86.11) | 1 |
| >=25 | 5 (16.13) | 5 (13.89) |  |
| Hypertension, n (%) |  |  |  |
| no | 30 (96.77) | 36 (100.00) | 0.94 |
| yes | 1 (3.23) | 0 (0.00) |  |
| Diabetes, n (%) |  |  |  |
| no | 31 (100.00) | 35 (97.22) | 1 |
| yes | 0 (0.00) | 1 (2.78) |  |

| **Supplementary table 3:**  Marker genes in 15 cell clusters. | |
| --- | --- |
| Celltype | Gene |
| Macrophage | *AC026369.3, FOLR3, PARAL1, MARCO, RETN, MCEMP1, FBP1, FABP4, FCGBP, TREM2* |
| Bone_cell | *MMP13, KLK4, ENPP1, SMOC1, WIF1, MEGF10, IBSP, BMP3, SPP1, C1QTNF1* |
| Chondrocyte | *AC074351.1, MYO15A, PAPPA2, HP, FGF10, SFRP2, CNMD, SCG2, GFRA1, COMP* |
| T_cell | *GZMH, NKG7, CD3G, GZMM, SKAP1, CD247, CTSW, GZMA, KLRB1, CD3E* |
| MSC | *UBE2C, SLC5A10, GTSE1, IL13RA2, CKAP2, PRAC1, TROAP, CDCA3, PCP4, TPX2* |
| Osteoclast | *DCSTAMP, SIGLEC15, AP000904.1, ACP5, OCSTAMP, SLC9B2, CTSK, RUFY4, ITGB3, ATP6V0D2* |
| Tissue_stem_cell | *RGS5, STMN2, SERPINE1, CA9, RGS4, SCUBE3, HHIP, PPP1R14A, GREM2, NPR3* |
| Endothelial_cell | *PLVAP, VWF, CLEC14A, CDH5, RAMP3, CXorf36, TM4SF18, KDR, MYCT1, ACKR1* |
| Neuron | *TAC3, COCH, CASC8, RP1, FAM166B, ZFP42, FABP7, KRTAP13-1, PTPRR, AC007207.2* |
| Skeletal_muscle_cell | *OSTN, HMX1, TNNC2, TP73, FAT2, LINC01896, RALYL, RBM46, LHX8, ESRRG* |
| Granulocyte | *IL17B, S100A1, NCAM2, MATN4, S100P, CDH19, CDH7, SLAMF9, CLEC3A, GRID2* |
| Monocyte | *LYZ, HLA-DRB5, JAML, LGALS2, CLEC10A, FCN1, FCER1A, S100A9, CD1C, HLA-DQA2* |
| Osteoblast | *JDP2, RUFY4, AP002807.1, OCSTAMP, NDRG4, NEURL3, F5, TXLNB, GALR1, AK8* |
| NK_cell | *C11orf96, CYGB, CCDC102B, ABCC9, SPON2, OLFML2A, KCNK17, KCNJ8, RASL12, POPDC2* |
| Treg | *FRS2, CCND2, BHLHE41, SSPN, LRIG3, HMGA2, CILP2, PTHLH, BGLAP, EXPH5* |

| **Supplementary table 4:**  Marker genes in 12 immune cell clusters. | |
| --- | --- |
| Celltype | Gene |
| Memory_T | *CD3D, CD69, PCP4, CD2, LTB, IL32, PRDX2, CD52, KLRB1, DPT* |
| Treg | *HLA-DRB5, PCP4, C1orf162, FCGR2B, DPT, CHN1, RGS2, PRDX2, CSTA, BEX3* |
| Monocyte | *ACKR3, HK2, LUCAT1, PLIN2, MARCO, SLC2A5, GDF15, PLA2G7, TAC3, ADAMDEC1* |
| NK | *IL32, CD7, CD3D, ITM2A, CD3E, CD2, NKG7, IL2RG, KLRB1, CD52* |
| mDC | *FCER1A, CD1C, CLEC10A, CFP, FCN1, LGALS2, CD300E, HLA-DQA2, S100A9, LYZ* |
| Neutrophil | *CCL18, S100A8, CXCL2, HLA-DRB5, CXCL3, BCL2A1, CXCL8, S100A9, C15orf48, NAMPT* |
| M2_macrophage | *FCGBP, RNASE1, HLA-DMB, AKR1B1, TREM2, C1QB, TMEM176B, VSIG4, FOLR2, STAB1* |
| M1_macrophage | *CCL2, IFI27, SPP1, IFI44L, FCGR1A, IFI6, LGALS3BP, VMO1, CD14, MMP13* |
| CD8+_T | *GZMH, CD8A, GZMM, GZMA, GZMK, LINC01871, SAMD3, PRF1, SKAP1, NKG7* |
| NKT | *NKG7, CD2, CCL5, GZMA, IL32, CD3D, CD52, IBSP, HLA-C, CD74* |
| pDC | *JCHAIN, GZMB, PLAC8, IRF7, AREG, C12orf75, TMEM107, TCF4, GPR183, HNRNPH1* |
| CD4+_T | *XCL1, GNLY, XCL2, TNFRSF18, KLRD1, COCH, TAC3, MEPE, CTSW, IL2RB* |

| **Supplementary table 5:**  Differential gene expression of immune checkpoint factors across various immune cell types between metastatic and primary osteosarcoma patients was observed. | | | |
| --- | --- | --- | --- |
| Celltype | Factor | p | Avg_log2FC |
| M1_macrophage | S100A8 | 0.003 | 3.557 |
| M2_macrophage | B7-H5 | 0.037 | 0.324 |
| M2_macrophage | CD47 | 0.007 | 0.254 |
| M2_macrophage | S100A8 | 0.003 | 1.578 |
| M2_macrophage | S100A9 | <0.001 | 2.584 |
| mDC | CD48 | 0.022 | 0.321 |
| mDC | TIMD-4 | <0.001 | 5.778 |
| mDC | CD47 | 0.012 | 0.429 |
| mDC | S100A8 | 0.005 | -5.086 |
| mDC | S100A9 | 0.012 | -3.329 |
| Memory_T | S100A9 | <0.001 | -4.905 |
| Monocyte | CD48 | 0.041 | 0.800 |
| Monocyte | TIMD-4 | 0.009 | 2.713 |
| Monocyte | S100A8 | <0.001 | 3.967 |
| Monocyte | S100A9 | 0.008 | 1.981 |
| Neutrophil | CD48 | <0.001 | 1.688 |
| Neutrophil | S100A8 | 0.019 | 1.037 |
| Treg | CD48 | <0.001 | -2.531 |
| Only cases with differences are shown here. | | | |

| **Supplementary table 6:**  Differential gene expression of immune checkpoint factors across various immune cell types between recurrent and primary osteosarcoma patients was observed. | | | |
| --- | --- | --- | --- |
| Celltype | Factor | p | Avg_log2FC |
| M1_macrophage | CD48 | <0.001 | 1.009 |
| M1_macrophage | B7-H6 | 0.002 | 3.091 |
| M1_macrophage | S100A8 | 0.019 | 1.465 |
| M2_macrophage | B7-H5 | 0.013 | 0.144 |
| Only cases with differences are shown here. | | | |

# Supplementary Figures

**Supplementary figure 1.** Bar plot of pathway enrichment for differential proteins (CD48, B7-H2, TIMD-4, B7-H6, CD134, B7-H5, CD47, S100A8, S100A9) generated by Metascape.


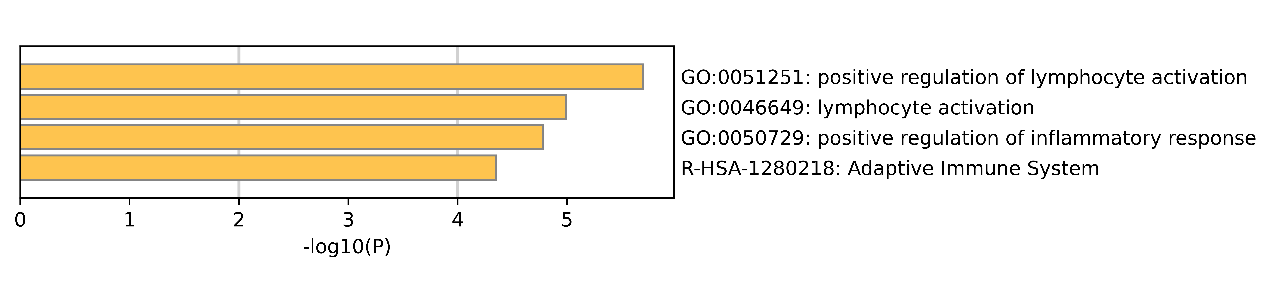


**Supplementary figure 2.** Additional metrics enhance the reliability of the clustering results. (A) Silhouette plot (K = 2). (B) Bar plot showing the distribution of optimal K values suggested by NbClust.


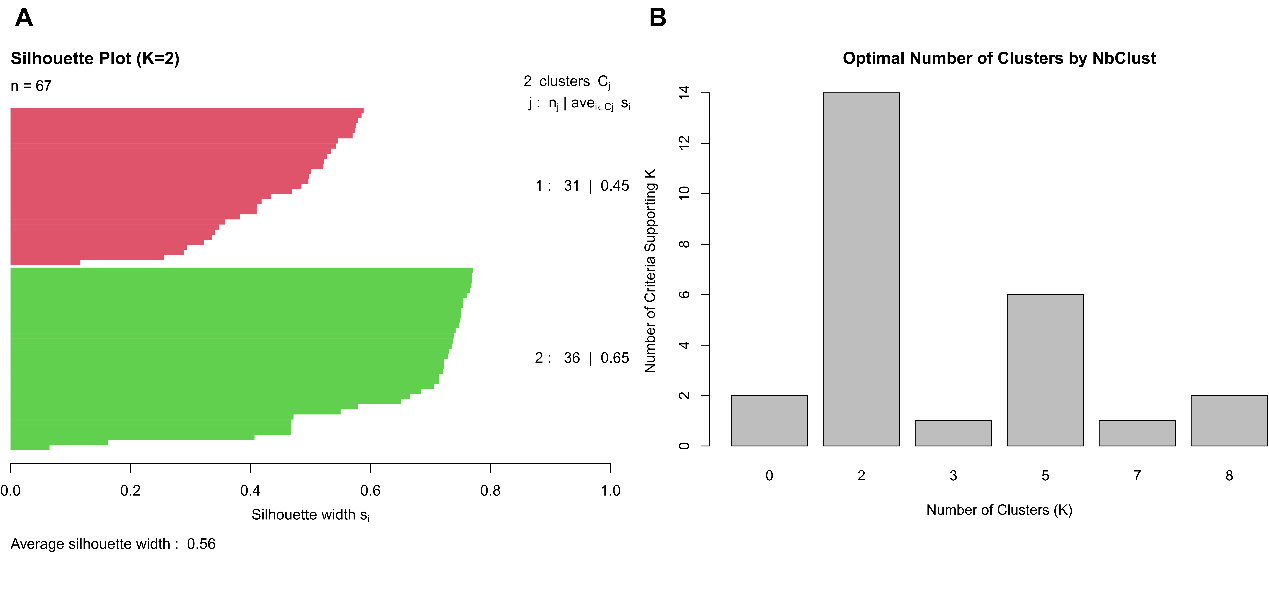


**Supplementary figure 3.** Decision curve analysis to evaluate net clinical benefit.


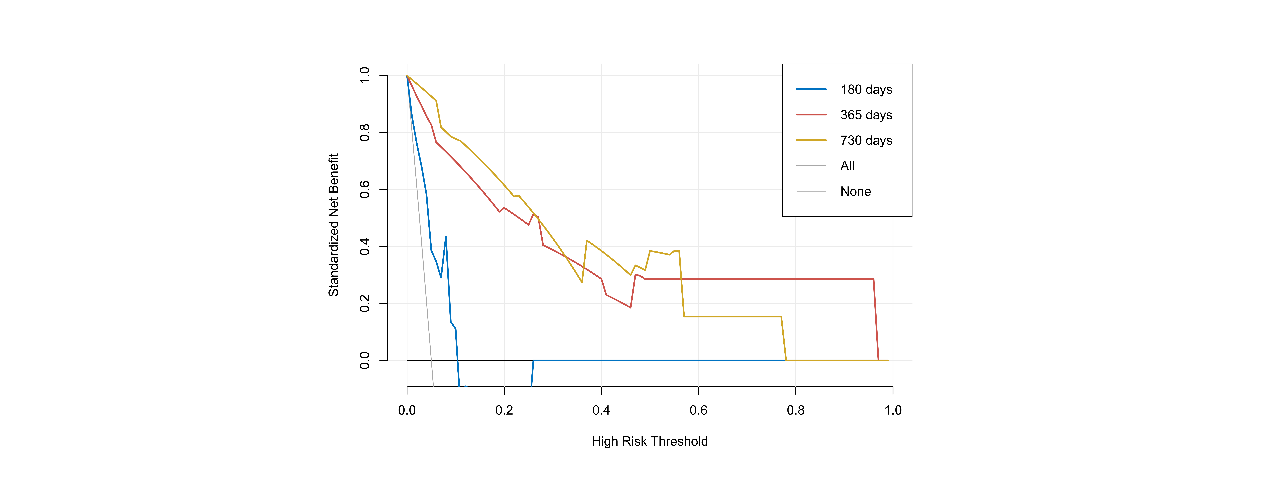


**Supplementary figure 4****.** Batch correction of scRNA-seq data. (A) UMAP plot before batch correction. (B) UMAP plot after batch correction.


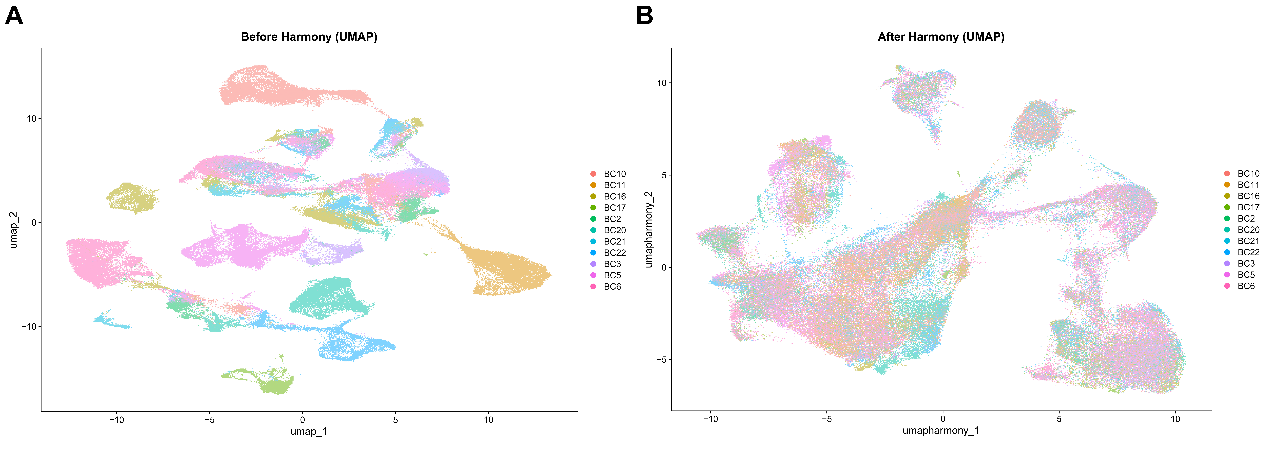


**Supplementary figure 5.** Single-cell transcriptomic analysis of osteosarcoma tumor tissues and overview of the tumor immune microenvironment. (A) t-Distributed Stochastic Neighbor Embedding (tSNE) plot of the major cell types identified in osteosarcoma tumor tissues. (B) tSNE plot of the immune cell types identified within the osteosarcoma tumor tissues. (C) tSNE plot displaying the expression levels of marker genes for each immune cell type, with red indicating high expression and gray indicating low expression. (D) Composition of immune cells in metastatic, primary, and recurrent osteosarcoma tumor tissues.


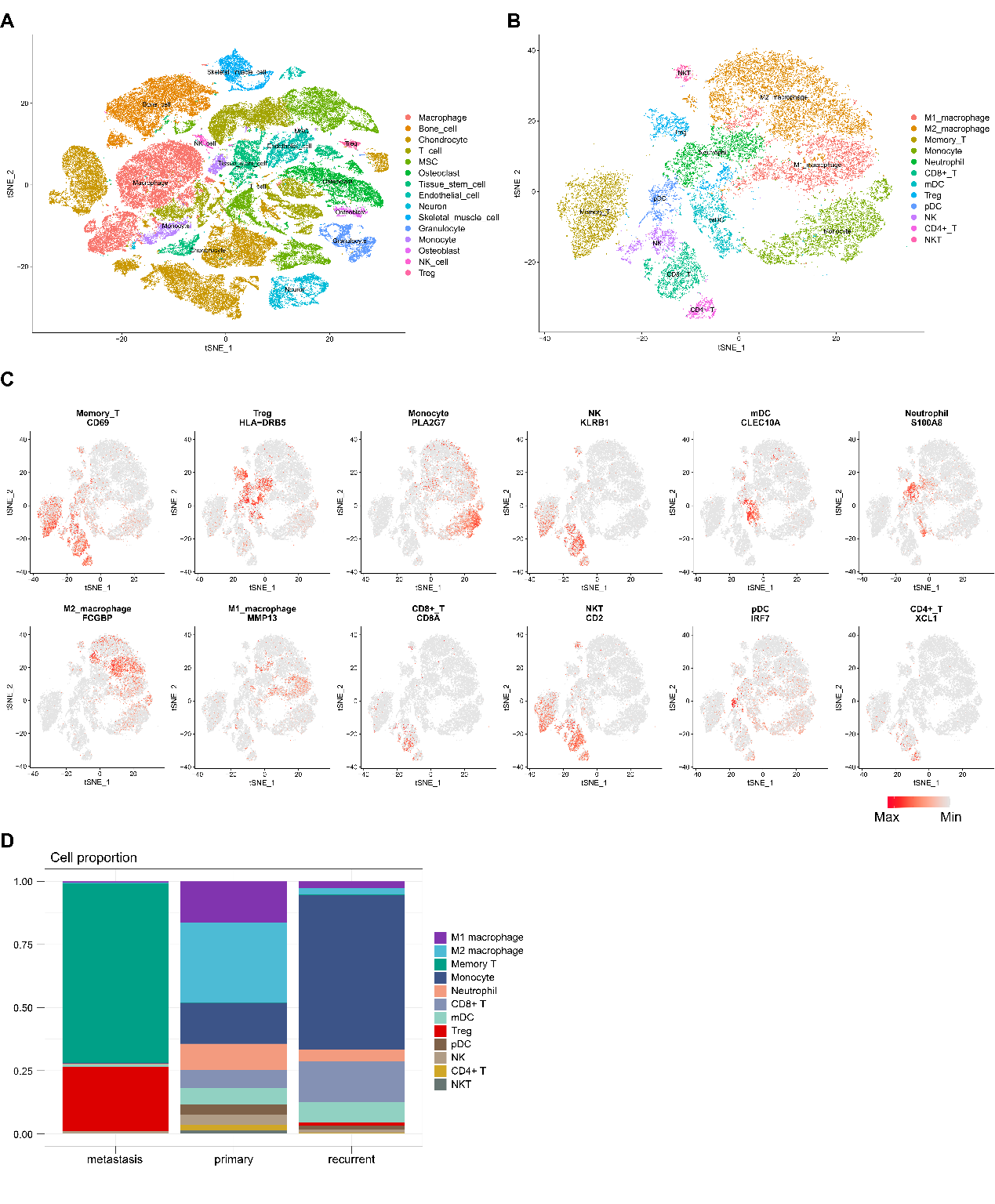

Supplement: Supplementary file 1 [file Table1.docx]
